# Vascular Access Type and Survival Outcomes in Hemodialysis Patients: A Seven-Year Cohort Study

**DOI:** 10.3390/medicina61040584

**Published:** 2025-03-25

**Authors:** Jesús Venegas-Ramírez, Gustavo A. Hernández-Fuentes, Claudia S. Palomares, Janet Diaz-Martinez, Joel I. Navarro-Cuellar, Patricia Calvo-Soto, Carlos Duran, Rosa Tapia-Vargas, Ana C. Espíritu-Mojarro, Alejandro Figueroa-Gutiérrez, José Guzmán-Esquivel, Daniel Antonio-Flores, Carmen Meza-Robles, Iván Delgado-Enciso

**Affiliations:** 1 Department of Nephrology, Mexican Institute of Social Security (IMSS), General Hospital of Zone No. 1, IMSS, Villa de Alvarez 28984, Mexico; nefrojesusvr@gmail.com; 2 Department of Molecular Medicine, School of Medicine, University of Colima, Colima 28040, Mexico; gahfuentes@gmail.com; 3 State Cancerology Institute of Colima, Health Services of the Mexican Social Security Institute for Welfare (IMSS-BIENESTAR), Colima 28085, Mexico; carmen.qfb@gmail.com; 4 Faculty of Chemical Sciences, University of Colima, Coquimatlan 28400, Mexico; 5 Department of Internal Medicine, Mexican Institute of Social Security (IMSS), General Hospital of Zone No. 1, Villa de Alvarez 28984, Mexico; selenepacr@gmail.com; 6 Department of Dietetics & Nutrition, Robert Stempel College of Public Health, Research Center in Minority Institutions, Florida International University, Miami, FL 33199, USA; jdimarti@fiu.edu; 7 Department of Research, Mexican Institute of Social Security (IMSS), General Hospital of Zone No. 1, Villa de Alvarez 28984, Mexico; rosagonzalez@ucol.mx; 8 Coordination of Planning and Institutional Liaison, IMSS OOAD Colima, Colima 28030, Mexico; patricia.calvo@imss.gob.mx; 9 Florida Kidney Physicians, Boca Raton, FL 33431, USA; cmartinez@flkidney.com; 10 Medical Education Auxiliary Coordination, Mexican Institute of Social Security (IMSS) OOAD Colima, Colima 28030, Mexico; rosa.tapia@imss.gob.mx; 11 Department of Pediatrics, Mexican Institute of Social Security (IMSS), General Hospital of Zone No. 1, Villa de Alvarez 28984, Mexico; aespiritu3@ucol.mx; 12 Health Education Auxiliary Coordination, Mexican Institute of Social Security (IMSS), Villa de Alvarez 28984, Mexico; alejandro.figueroag@imss.gob.mx; 13 Clinical Epidemiology Research Unit, Mexican Institute of Social Security (IMSS), Villa de Alvarez 28984, Mexico; jose.esquivel@imss.gob.mx; 14 Department of Nephrology, Mexican Institute of Social Security (IMSS), General Hospital of Zone No. 10, Manzanillo 28869, Mexico; restiforme@gmail.com; 15 Robert Stempel College of Public Health and Social Work, Florida International University, Miami, FL 33199, USA

**Keywords:** hemodialysis, arteriovenous fistula, central venous catheter, survival analysis, cohort study, mortality risk, patient-centered care

## Abstract

*Background and Objectives*: Arteriovenous fistulas (AVFs) are the preferred vascular access for hemodialysis due to their impact on patient outcomes, including survival, infection rates, and overall quality of life. Despite strong recommendations favoring AVF, gaps in AVF utilization remain, influenced by clinical, demographic, and systemic factors. This study is the first to analyze survival outcomes associated with different dialysis vascular access types, adjusting for key clinical, demographic variables, and other comorbidities over extended periods. *Materials and Methods*: This ambispective cohort study followed 428 hemodialysis patients over seven years. Patients were categorized based on their access type: AVF (*n* = 189), tunneled central venous catheter (CVC) (*n* = 178), and non-tunneled CVC (*n* = 61). Kaplan–Meier survival analysis was used to estimate survival curves, and Cox proportional hazards regression adjusted for potential confounders, including age, diabetes, and hypertension. *Results*: The 2-year survival rates were as follows: AVF 94.1%, tunneled CVC 70.0%, and non-tunneled CVC 36.6%. The 7-year survival rates were as follows: AVF 65.5%, tunneled CVC 26.4%, and non-tunneled CVC 11.0%. Compared to AVF, tunneled CVC use was associated with a 2.8-fold increased risk of mortality (adjusted hazard ratio [AdHR] 2.8, 95% CI 2.0–4.1), while non-tunneled CVC increased the risk 5-fold (AdHR 5.0, 95% CI 3.3–7.6). Notably, older adults, women, and diabetic patients were disproportionately represented in the groups with tunneled and non-tunneled catheters. *Conclusions*: Adjusted survival analyses highlight the significantly lower survival rates associated with CVC use compared to AVF. Non-tunneled catheters are generally not used for prolonged periods, and this cohort provides evidence of their prognosis for long-term use. These findings reinforce the need to prioritize AVF placement whenever feasible, reinforcing health education on this topic, to improve long-term outcomes for hemodialysis patients.

## 1. Introduction

Chronic kidney disease (CKD) is a growing global public health concern, affecting more than 10% of the global population, with an estimated 800 million individuals living with the condition. CKD is progressive, and a significant proportion of patients advance to end-stage renal disease (ESRD), which requires renal replacement therapy such as hemodialysis (HD) to sustain life [1,2]. Globally, HD is the predominant modality of renal replacement therapy, utilized by 70% to 90% of ESRD patients, including nearly half a million individuals in the United States alone [1]. In Mexico, hemodialysis is the primary renal replacement therapy for patients with end-stage renal disease (ESRD), with a significant proportion relying on publicly funded healthcare institutions such as the Mexican Institute of Social Security (IMSS) and the Institute of Security and Social Services for State Workers (ISSSTE), as well as private providers [1,2]. IMSS alone provides renal replacement therapy to approximately 79,689 patients. However, access to hemodialysis services is often limited by geographic, economic, and infrastructural barriers, which results in disparities in treatment and outcomes [3,4]. The prevalence of ESRD in Mexico has been increasing, mainly due to high rates of diabetes and hypertension, the leading causes of chronic kidney disease (CKD) in the country [5].

Vascular access is crucial for HD patients, and the type of access plays a significant role in patients’ outcomes, including survival and infection rates. There are two main types: arteriovenous (AV) access and central venous catheters (CVCs). AV access includes arteriovenous fistulas (AVFs) and arteriovenous grafts (AVGs), both of which are preferred over CVCs due to their superior long-term patency and lower complication rates [6,7]. Arteriovenous grafts (AVGs), however, have limited use in low- and middle-income countries. For example, in Mexico, recent studies showed that only 1.5% of patients used them, highlighting their rare utilization in clinical practice [8]. Despite strong recommendations favoring AVF as the preferred access, disparities in vascular access selection persist, influenced by clinical, demographic, and systemic factors [3,4].

AVF is considered the gold standard due to its superior long-term patency, significantly lower rates of thrombosis and infection, and reduced healthcare-related expenditures compared to AVG and CVC [5,6]. When successfully matured, AVF offers the best long-term outcomes for most HD patients, minimizing complications such as hospitalization and mortality [6,9]. Despite these well-documented advantages, their use remains suboptimal in many patient populations. CVCs, both tunneled and non-tunneled, are often employed as alternatives, particularly in patients with advanced age, multiple comorbidities, or anatomical challenges that may complicate fistula creation [10]. However, these catheters are associated with higher risks of bloodstream infections, thrombosis, and hospitalization, leading to poorer clinical outcomes [11,12]. In the United States, approximately 80% of patients initiate HD with a CVC [13,14]. In emergent or urgent dialysis situations, non-tunneled CVCs serve as a non-tunneled solution for managing conditions such as intoxications, uremic symptoms, or volume overload. Tunneled CVCs, most commonly placed in the internal jugular vein under imaging guidance, are preferred for longer-term use, ensuring the catheter tip resides in the mid-right atrium. Non-tunneled CVCs, inserted under ultrasound guidance, should be used only for short durations (up to two weeks) to minimize the risk of catheter-related infections. While CVCs are generally not ideal for long-term vascular access, they may be used tunneled in select cases when other options are not feasible [6,11].

International guidelines, such as the National Kidney Foundation–Kidney Disease Outcomes Quality Initiative (NKF-KDOQI) and the Fistula First Breakthrough Initiative in the United States (US), recommend prioritizing the use of AVFs over AVGs and aim to reduce reliance on CVCs [6,7]. Consequently, AVF use among long-term dialysis patients in the US increased from 32% in 2003 to 65% in 2014, while CVC use has concurrently declined [14]. However, despite these efforts, 80% of incident dialysis patients still initiate treatment with a catheter, and only one-quarter of these patients have a maturing AVF or AVG at the time of dialysis initiation [14]. While the United States has made progress in promoting AVF use [13,14], global disparities in vascular access utilization and success rates remain pronounced [10]. Findings from the Dialysis Outcomes and Practice Patterns Study (DOPPS) reveal substantial variations in AVF prevalence, ranging from over 90% in countries like Japan and Russia to just 49% in other regions [11]. Similarly, CVC dependence varies widely, with rates spanning 1% to 45% across different countries [12]. In Mexico, vascular access for hemodialysis varies significantly, with patients utilizing arteriovenous fistulas (AVFs), tunneled catheters, and non-tunneled catheters depending on healthcare availability and individual clinical conditions [12]. While AVFs are the preferred option due to their lower risk of infection and longer durability, their use remains suboptimal due to late nephrology referrals, limited surgical expertise, and delays in vascular access planning. These global differences in vascular access use reflect variations in healthcare infrastructure, surgical expertise, early CKD care, and vascular access planning, all of which continue to shape patient outcomes [15].

Beyond systemic and structural disparities, patient-level factors also play a crucial role in vascular access selection and outcomes [7]. Education, perceived benefits, and aesthetic concerns influence a patient’s willingness to undergo AVF placement [16]. Barriers such as low health literacy, lack of disease awareness, and misperceptions about AVFs can further deter their acceptance [17]. Additionally, previous studies suggest that comorbidities such as diabetes and hypertension may significantly impact vascular access choice for HD [11,14]. Following AVF creation, several factors contribute to maturation failure, including age, vessel diameter, blood flow, and comorbidities like diabetes and cardiovascular disease [15]. The interplay between these physiological and patient-related factors underscores the complexity of vascular access selection across different patient populations, yet epidemiological studies on this subject remain scarce.

This study examines the distribution of vascular access types in a cohort of Mexican hemodialysis patients and analyzes the demographic and clinical characteristics associated with access selection and its implications for survival. It explores how factors, such as age, sex, diabetes, and hypertension, influence vascular access choice and mortality risk. By providing a deeper understanding of the factors shaping vascular access selection and related outcomes, this research contributes to the broader discourse on patient-centered hemodialysis care, optimization of vascular access practices, and improved clinical outcomes.

## 2. Materials and Methods

### 2.1. Study Design and Setting

This ambispective cohort study was conducted between 2018 and 2023 at a single tertiary care center specializing in hemodialysis (General Hospital Zone 1 of the Mexican Social Security; Colima, Mexico). The study followed patients over a maximum period of 7 years to evaluate the association between vascular access types and long-term survival. The three vascular access types included arteriovenous (A-V) fistulas, tunneled catheters, and non-tunneled catheters. Relevant clinical risk factors were adjusted to provide a comprehensive analysis of patient outcomes.

### 2.2. Participants

**Figure 1:**
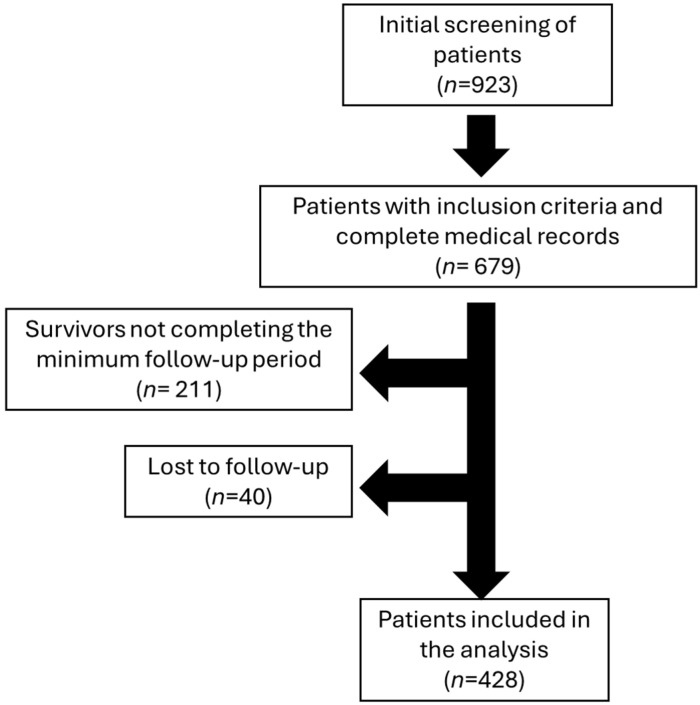
Flowchart of patient selection and characteristics for hemodialysis analysis.

This study included adult patients undergoing hemodialysis, selected based on specific inclusion and exclusion criteria. Participants were eligible for inclusion if they were 18 years of age or older and received regular hemodialysis treatment using one of the predefined vascular access types (arteriovenous fistula, tunneled catheter). Patients with AVGs were not included, as this type of vascular access is rarely used in the analyzed healthcare institution (only four patients during the study period). Those with incomplete clinical records were excluded, as were individuals lost to follow-up before the study’s conclusion. Careful screening ensured that the final cohort comprised only patients with comprehensive data and consistent follow-up, enhancing the reliability of the study findings. Additionally, patients who survived during the follow-up period but had less than seven years of follow-up were excluded, along with those who were lost to follow-up. A total of 428 patients were included in the analysis after excluding 211 living patients who did not complete the minimum follow-up period and 40 patients lost to follow-up (28 with tunneled catheters, 11 with AVFs, and 1 with a temporary catheter) (see Figure 1).

### 2.3. Variables

The primary exposure variable in this study was the type of vascular access used for hemodialysis, which included arteriovenous fistulas, tunneled catheters, and non-tunneled catheters. An arteriovenous fistula (AVF) is a surgically created connection between an artery and a vein, usually in the arm, and is considered the gold standard for vascular access. AVFs are preferred due to their superior long-term patency, lower risks of infection and thrombosis, and their association with improved survival rates [18]. Tunneled catheters, on the other hand, are tunneled devices inserted into large central veins, such as the internal jugular or subclavian vein, and are designed for long-term use [19]. Although they are a viable alternative for patients who cannot undergo AVF creation, tunneled catheters carry significantly higher risks of bloodstream infections, thrombosis, and other complications. Non-tunneled catheters are typically placed for short-term use and are inserted percutaneously into central veins [20]. While they are often used as an immediate solution for vascular access, non-tunneled catheters are associated with the highest risks of infection, thrombosis, and adverse outcomes, making them unsuitable for long-term hemodialysis. The primary outcome of this study was overall survival (OS) over a 7-year follow-up period. Covariates included demographic and clinical factors, such as age, gender (male or female, based on official identification), diabetes mellitus (diagnosed based on medical history or the use of hypoglycemic agents), and hypertension (defined as a history of blood pressure > 140/90 mmHg or the use of antihypertensive medication). Other covariates included hospitalization (defined as any hospital admission lasting more than 24 h), sepsis (documented systemic infection requiring antimicrobial treatment during follow-up), myocardial infarction (MI) (diagnosed based on clinical, biochemical, and electrocardiographic criteria), and cerebrovascular disease (CVD) (defined as a history of ischemic or hemorrhagic stroke confirmed by imaging or clinical evaluation).

### 2.4. Data Collection

Patient data were collected from the institutional electronic medical records, where demographic, clinical, and laboratory information was extracted. This included details about the type of vascular access, comorbidities, and patient outcomes, such as mortality and hospitalizations. To ensure accuracy, the survival status of all patients was verified through hospital records, and when necessary, follow-up was conducted via telephone.

### 2.5. Ethical Considerations

The study protocol was reviewed and approved by the local health research committee of IMSS-Colima, Mexico, under approval number R-2018-785-056 on 12 June 2018. Informed consent was waived as the data were obtained retrospectively from clinical records and not directly from patients. All data were anonymized to protect patient confidentiality. This study adhered to the ethical principles outlined in the Declaration of Helsinki, maintaining strict confidentiality and data security throughout the research process [21].

### 2.6. Sample Size and Power Calculation

The sample size was estimated based on the survival differences observed between vascular access types over a 2-year period [22], as reported in previous studies [6], where survival with an AVF was 69%, compared to 38% with a catheter. Using a significance level (α) of 0.05 and a statistical power (1-β) of 80%, the required sample size for comparing the groups with the largest survival contrasts was calculated to be 39 patients per group. Upon completion of this study, a post hoc statistical power analysis was conducted. The analysis revealed that having an AVF significantly reduced mortality in hemodialysis patients after 7 years of follow-up. The statistical power for this finding was 99.9% when compared to the tunneled catheter group and 99.9% when compared to the non-tunneled catheter group.

### 2.7. Statistical Analysis

Continuous variables were reported as means with their corresponding standard deviations (SD), while categorical variables were summarized as counts and percentages. Group comparisons were performed using Fisher’s exact test for qualitative variables, while ANOVA or Student’s *t*-test was used for quantitative data, as appropriate [23]. The normality of the data distribution was verified using the Kolmogorov–Smirnov test. Survival outcomes over a 7-year period were assessed for a cohort of 428 patients using the Kaplan–Meier method for survival analysis. To identify independent risk factors for overall survival, univariate and multivariate Cox proportional hazards regression analyses were conducted, generating hazard ratios (HRs), which were interpreted as measures of relative risk [24]. The predictors of significant outcomes were determined and subsequently examined in a multivariable model. A forward stepwise Cox regression was then used to identify the most efficient model. For the stepwise regression, a variable entry threshold of 0.05 and a removal threshold of 0.10 were applied. The vascular access route (AVF, tunneled catheter, or non-tunneled catheter) was incorporated into the model as a ‘stratum’ only when analyzing the risk of covariates. The Omnibus Tests of Model Coefficients were used to evaluate whether the inclusion of explanatory variables significantly improved the baseline survival model. A chi-square test was employed to compare the log-likelihoods of the baseline and updated models, assessing significant differences [25]. The concordance index (C-index) was calculated to assess the predictive ability of the multivariate Cox regression models. The C-index was calculated using R software (ver. 4.4.1). The adjusted survival curve, derived from the Cox Proportional Hazards Model, was graphically displayed, representing the survival function at the mean of the covariates [25,26]. All statistical analyses were conducted using SPSS version 25 (IBM Corp., Armonk, NY, USA), except for statistical power and sample size, which were calculated using ClinCalc version 1 (https://clincalc.com/stats/Power.aspx, accessed on 15 January 2025) [27,28]. A significance level of *p* < 0.05 was considered statistically significant.

## 3. Results

### 3.1. General Characteristics

Initially, 923 patients were screened, of whom only 679 were included because they had a complete medical record. After the evaluation period was over, 211 survivors were eliminated because they did not complete the minimum follow-up period required, which was 7 years. Forty patients were lost to follow-up. A total of 428 patients on hemodialysis were included in the analysis (Figure 1), with the majority having a tunneled catheter (53.0%, *n* = 227), followed by AVF (27.8%, *n* = 119) and non-tunneled catheter (19.2%*, n* = 82). The average age was 58.39 years (SD 15.53), with 38.6% of patients being women. Age was significantly lower in patients with AVF (51.75 years, SD 15.59) compared to those with a tunneled catheter (60.78 years, SD 14.99, *p* < 0.001) or non-tunneled catheter (61.43 years, SD 14.13, *p* < 0.001).

Table 1 shows that women, patients aged 60 years or older, and those with diabetes were significantly more likely to have a tunneled or non-tunneled catheter, compared to those with an AVF (*p* < 0.05 for all comparisons, see Table 1). It can be observed that patients with AVFs had fewer occurrences of sepsis and hospitalizations (see Table 1). Notably, after 7 years of follow-up, the group with AVF had the lowest mortality rate (34.5%), which was significantly lower than the mortality rates of those with a tunneled catheter (73.6%, *p* < 0.001) and non-tunneled catheter (89.0%, *p* < 0.001) (see Table 1).

### 3.2. Effect of Vascular Access Types on Survival

**Figure 2:**
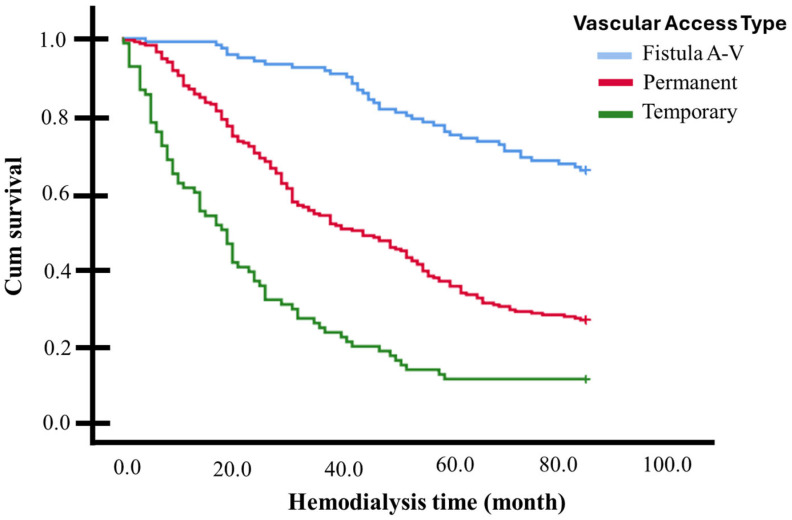
Kaplan–Meier curves for 84-month follow-up (7 Years) based on vascular access for dialysis: individuals with an AVF exhibited a higher survival rate than those with tunneled and non-tunneled catheters (*p* < 0.001, multigroup log-rank test).

In a 7-year (84 months) survival follow-up (Kaplan–Meier curves), significant differences were observed among the various vascular access types for dialysis (*p* < 0.001, multigroup log-rank test). Individuals with an AVF exhibited a higher survival rate (mean 72.4 months, 95% CI 68.7–76.2, survivors 65.5%), while those with a non-tunneled catheter had a lower survival rate (mean 25.9 months, 95% CI 20.4–31.4, survivors 11.0%), with individuals with a tunneled catheter falling in between (mean 46.8 months, 95% CI 43.1–50.6, survivors 26.4%) (see Figure 2 and Table 2).

### 3.3. Adjusted Overall Survival According to the Presence of Other Risk Factors

The overall survival (OS) information based on the Cox Proportional Risk Model is provided in Table 3 and Table 4. The independent variables included in the model are as follows: age (>60 years), gender, history of diabetes, hypertension, hospitalization, sepsis, acute myocardial infarction (AMI), and stroke (EVC). Table 3 shows the collective controls for adjusting the reciprocal model. Since the χ^2^ variation is statistically significant (*p* < 0.001), the model is well adjusted, meaning that at least one of the independent variables significantly affects survival time (see Table 3).

**Figure 3:**
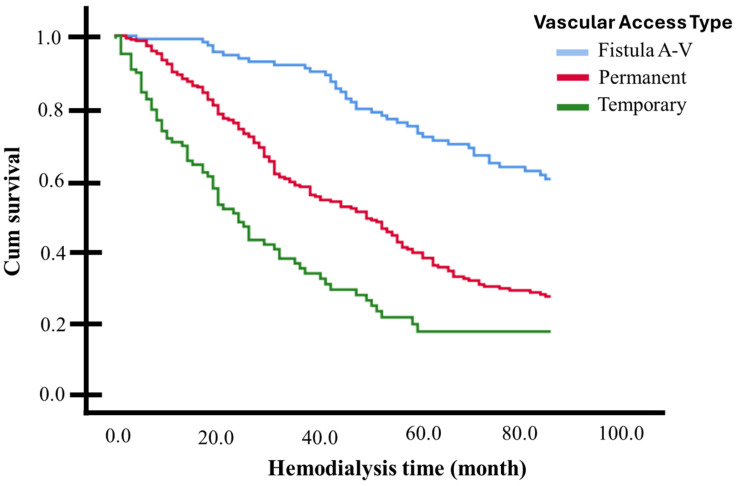
Adjusted survival curves for patients with or without significant risk factors identified. Log-rank test (*p* < 0.05).

Table 4 presents the hazard ratios (HRs) obtained from the univariate and multivariate Cox regression models. The types of vascular access for hemodialysis were all considered as strata. Across 84 months (7 years) of hemodialysis, being diabetic (AdHR 2.38, 95% CI 1.77–3.20) or having been hospitalized (AdHR 1.80, 95% CI 1.36–2.38) approximately doubles the risk of death, while having a history of hypertension reduces this risk by half (AdHR 0.45, 95% CI 0.34–0.60). Being 60 years or older increases the likelihood of death by 37% during the follow-up period compared to those younger than 60 years (AdHR 1.374, 95% CI 1.052–1.795). Since these three factors frequently occur together, interactions between them were analyzed in independent multivariate models. The joint presence of diabetes and hypertension nullified the risk modification presented by each of these variables separately (AdHR 1.218, 95% CI 0.949–1.562, *p* = 0.122), as did having hypertension and being 60 years or older (AdHR 1.067, 95% CI 0.828–1.373, *p* = 0.617). Simultaneously having diabetes and being 60 years or older (AdHR 2.006, 95% CI 1.574–2.557, *p* < 0.001), or having diabetes and being hospitalized (AdHR 2.215, 95% CI 1.633–3.004, *p* < 0.001), or being 60 years or older and hospitalized (AdHR 1.825, 95% CI 1.309–2.544, *p* < 0.001), did not substantially modify the risk generated by diabetes or hospitalization alone. The protective factor of having a history of hypertension disappears in patients who have been hospitalized, with an elevated risk of death when these two factors occur together (AdHR 1.567, 95% CI 1.187–2.069, *p* = 0.002). A statistical model that included all the adjustment variables listed in Table 4 determined that having a tunneled catheter increased the probability of death 2.8-times in the 7-year follow-up period, compared with AVF (AdHR 2.892, 95% CI 2.036–4.108, *p* < 0.001). On the other hand, the use of a non-tunneled catheter increased the probability of death 5-times compared with AVF (AdHR 5.047, 95% CI 3.333–7.643, *p* < 0.001). In terms of model performance, the concordance index (C-index) for the tunneled catheter model was 0.684, reflecting reasonable discriminatory ability to differentiate between patients who experienced death and those who did not over the 7-year follow-up period. In comparison, the C-index for the non-tunneled catheter model was 0.768, suggesting a slightly better discriminatory ability. These values indicate that both models demonstrate good overall predictive accuracy. The adjusted survival curve (based on the Cox Proportional Risk Model) is graphically presented in Figure 3.

### 3.4. Survival Based on the Presence or Absence of Risk Factors

**Figure 4:**
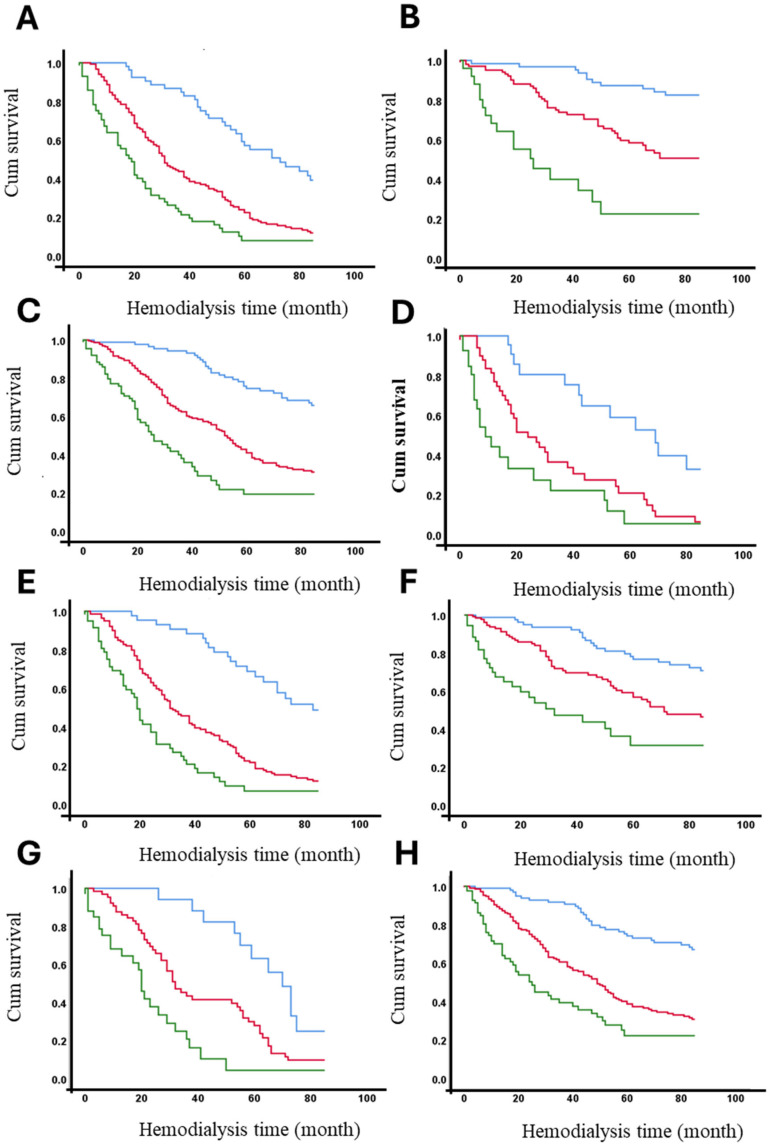
Adjusted survival curves (in months) based on vascular access and the presence or absence of significant risk factors. Panels represent survival stratified by different conditions: (**A**) diabetic patients, (**B**) non-diabetic patients, (**C**) hypertensive patients, (**D**) non-hypertensive patients, (**E**) patients aged ≥ 60 years, (**F**) patients aged < 60 years, (**G**) patients with hospitalization, and (**H**) patients without hospitalization. Vascular Access Type: AVF (blue line), Permanent (red line) and Temporary (green line).

The 7-year survival rates of hemodialysis patients were analyzed according to the presence or absence of identified risk factors (see Table 3). Table 5 demonstrates that age >60 years, diabetes, hypertension, and hospitalization significantly impact survival rates across the entire cohort, particularly in patients with AVFs and tunneled catheters (see Table 4). However, in patients with non-tunneled catheters, no significant differences in 7-year survival rates were observed based on these risk factors, likely due to the low frequency of patients surviving this period. Across the entire cohort, only 18.4% of diabetic patients survived 7 years, compared to 58.1% of non-diabetic patients. Vascular access type significantly influenced these outcomes. Among patients with AVFs, 41.2% of diabetics and 83.8% of non-diabetics survived (*p* < 0.001). In contrast, among patients with non-tunneled catheters, only 8.2% of diabetics and 19.0% of non-diabetics survived (*p* < 0.001). Detailed survival percentages for all strata are provided in Table 4. Figure 4 presents the adjusted survival curves for patients with or without each of the significant risk factors identified in this study.

The survival function at the mean of covariates is presented according to vascular access type and the presence or absence of risk factors that significantly affect survival, as shown in Table 3 and Table 4. The curves demonstrate that being diabetic (A), not having hypertension, being aged 60 years or older, and having been hospitalized are factors associated with reduced survival over time. The Figure 4 clearly illustrates the cumulative impact of these factors on overall survival, highlighting the differences across vascular access types and patient risk profiles.

To identify interactions between variables, we analyzed the stratum-specific effects of various factors. Effect modification, which involves stratification, occurs when an exposure produces different effects across subgroups. This approach includes visually inspecting the data to identify patterns of variation in stratum-specific estimates. If these estimates remain consistent, it suggests no interaction, whereas discrepancies indicate the presence of an interaction, highlighting how the association of interest is influenced by the stratifying factor. It is important to note that while stratum-specific effect modification is useful for exploring and suggesting potential interactions, effect modification can occur without interaction, and interaction can exist without effect modification [29,30,31].

We conducted a stratified analysis by categorizing the study population into subgroups based on significant clinical variables identified in the prior multivariate analysis (Table 4). This method enabled the investigation of effect modification (AdHR) on mortality, with AVF serving as the reference category (see Table 6). It can be observed that a modification effect exists, meaning changes in the AdHR based on the presence or absence of being 60 years or older, hypertension, diabetes, or a history of hospitalization. This pertains to the increased mortality associated with having a tunneled or non-tunneled catheter compared to AVF (reference). A significant difference is highlighted between the diabetes strata, where having a non-tunneled vascular access increases the probability of death by 10-times over 7 years compared to AVF (AdHR 10.024, 95% CI 4.351–23.098), whereas in diabetic individuals, the probability of death was only 4-times higher compared to AVF (AdHR 4.418, 95% CI 2.752–7.093) (Table 6).

## 4. Discussion

This study provides a comprehensive evaluation of the long-term impact of vascular access type on overall survival among hemodialysis (HD) patients, with a seven-year follow-up period. To our knowledge, this is one of the most extensive studies to analyze survival differences among patients using arteriovenous fistulas (AVFs), tunneled catheters, and non-tunneled catheters, while accounting for comorbidities and clinical risk factors in a high-risk population. Our findings demonstrate that patients with AVFs had the highest survival rates, with a 65.5% survival rate at seven years, compared to 26.4% for tunneled catheter users and 11.0% for non-tunneled catheter users. The use of both non-tunneled and tunneled catheters was associated with significantly reduced survival rates compared to AVFs, reinforcing their limited suitability for long-term HD.

These results align with global guidelines and recommendations, which emphasize the critical role of optimal vascular access in determining long-term survival among patients undergoing HD [32]. In addition, our findings also highlight the modulating effects of clinical and demographic factors on vascular access outcomes, particularly age, diabetes, hypertension, and hospitalization. Consistent with prior studies, diabetes and hospitalization were strongly associated with an increased mortality risk [6], whereas hypertension appeared to have a protective effect when considered in isolation. This seemingly paradoxical finding aligns with the concept of “reverse epidemiology” in dialysis patients, where traditional cardiovascular risk factors, such as hypertension and higher body mass index, have been linked to improved survival outcomes in this population [33,34]. Several physiological mechanisms have been proposed to explain this phenomenon, including the potential role of higher blood pressure in maintaining adequate perfusion of the arteriovenous fistula (AVF) and preventing access failure. Additionally, hypertension has been associated with a higher likelihood of successful AVF maturation, which could contribute to improved long-term vascular access patency and, consequently, better survival outcomes [35]. However, while hypertension may appear protective in this context, it is important to interpret these findings with caution. The long-term effects of poorly controlled hypertension on cardiovascular health, arterial stiffness, and vascular complications remain a concern [36]. Further research is needed to better understand the balance between its potential short-term benefits and long-term risks in hemodialysis patients [37]. These observations underscore the complex interplay between patient comorbidities and vascular access outcomes, emphasizing the need for individualized vascular access planning in high-risk populations with multiple comorbidities.

Interestingly, the survival advantage conferred by AVF remained robust even in the presence of adverse risk factors, including diabetes and advanced age. The protective effect of AVF persisted after adjusting for multiple risk factors, as demonstrated by Cox proportional hazards modeling. The survival benefits associated with AVF were consistent across all subgroups, even among patients with significant comorbidities such as diabetes. For instance, among diabetic patients, those with AVF had a survival rate of 41.2% after seven years, compared to 14.6% for those with tunneled catheters and 8.2% for those with non-tunneled catheters. These findings emphasize the importance of prioritizing AVF placement, particularly in high-risk populations such as diabetic patients. However, our study also revealed that the survival advantage of AVF was less pronounced in older adults, especially when combined with diabetes or frequent hospitalizations [32]. This suggests that the benefits of superior vascular access may be partially offset by comorbidities and complications associated with aging, highlighting the need for individualized treatment plans in older HD patients. In contrast, the already-poor survival outcomes associated with tunneled and non-tunneled catheters were further exacerbated by risk factors such as diabetes and advanced age. An important factor that may influence the mortality of patients with CVC is catheter-related infection. A recent meta-analysis examined the impact of managing these infections and suggested that appropriate treatment could improve survival [38]. While reduced Kt/V is generally associated with higher mortality, infections may also play a crucial role in patient outcomes. Therefore, proper infection management should be considered in future studies and clinical strategies to enhance patient survival [38].

These findings reinforce the urgent need to minimize catheter use and to transition patients with tunneled catheters to AVF whenever feasible, particularly in those with comorbid conditions that further compromise survival. The survival advantage of AVF observed in this study aligns with prior research, which has consistently established AVF as the preferred vascular access for hemodialysis due to its lower risks of complications, including infections, thrombosis, and other adverse events [17,25,26,39]. Conversely, the high mortality rates observed in patients with tunneled and non-tunneled catheters reflect the significant risks associated with catheter use, particularly catheter-related bloodstream infections (CRBSIs) and poor hemodynamic performance [28,32,40].

These findings have significant implications for clinical practice and health policy. They reinforce KDOQI recommendations to prioritize AVF as the preferred vascular access for chronic HD patients [40] and emphasize the need for early referrals to vascular surgery to ensure timely fistula creation, particularly in high-risk patients such as those with diabetes. This study also underscores the urgency of minimizing catheter use and transitioning patients to AVF whenever possible, especially those with comorbidities that compromise survival. Additionally, the observed interplay between comorbidities and vascular access type highlights the need for a patient-centered approach in vascular access planning.

Beyond vascular access type, this study identified several independent clinical predictors of survival. Diabetes mellitus increased the mortality risk by 2.38-times, while hospitalizations elevated it by 1.80-times, consistent with previous studies. Advanced age (≥60 years) was also associated with a 37% higher mortality risk. Interestingly, hypertension, traditionally considered a cardiovascular risk factor, appeared to have a protective effect, reducing the mortality risk by 55%. This aligns with the concept of “reverse epidemiology” observed in dialysis populations, where higher blood pressure may confer survival benefits under certain clinical conditions [35,39]. Given the well-established impact of diabetes on mortality in HD patients [41], addressing modifiable risk factors, such as diabetes control and infection prevention, remains essential for improving long-term outcomes in this high-risk population [26].

The analysis of our data raises critical concerns about potential disparities in access to optimal vascular modalities, particularly AVF placement. Older adults, women, and diabetic patients were disproportionately represented in the groups with tunneled and non-tunneled catheters, while AVF recipients were significantly younger, with a mean age of 51.75 years, compared to 60.78 years for tunneled catheter users and 61.43 years for non-tunneled catheter users. These patterns may suggest the presence of implicit biases or structural barriers that may inadvertently disadvantage certain populations, preventing them from accessing the survival benefits associated with AVF. Zarkowsky et al. (2015) [42] also reported that being female, of Black race, or having peripheral arterial disease reduced the probability of starting hemodialysis with an AVF. Additionally, their findings indicated that hypertension was linked to a higher likelihood of successful AVF maturation at the initiation of dialysis. Like our study, they found that diabetes negatively impacted AVF maturation.

Several other factors may contribute to these disparities. Physicians may prioritize AVF placement in younger patients, assuming a better long-term prognosis, which could unintentionally disadvantage older adults [34]. Similarly, anatomical differences in women or higher surgical risks in diabetic patients may influence clinical decision making, despite evidence supporting the long-term benefits of AVF in these populations [40,41]. Additionally, socioeconomic barriers, limited access to specialized care, and inadequate patient education about vascular access options may further contribute to these inequities [13].

It is essential to recognize the barriers at the patient, physician, and systemic levels that hinder timely referral for preoperative vein mapping, AVF placement, and maturation. These challenges are often exacerbated by delays in nephrology care before dialysis initiation [13,14]. Additionally, the unpredictable progression of CKD to ESRD and the variability in dialysis initiation thresholds further complicate timely vascular access planning. Some patients may also experience denial or reluctance to undergo vascular placement, opting instead for immediate but suboptimal solutions, such as tunneled and non-tunneled catheters, which prioritize clinical expediency over long-term benefits [43,44].

Addressing these challenges, particularly for older adults, women, and diabetic patients, requires early nephrology referral, structural interventions, and comprehensive patient education to ensure equitable access to optimal vascular care. All patients, regardless of demographic or clinical factors, must be informed about the survival and outcome advantages of AVF, enabling them to make informed decisions about their treatment. CKD education during stages 4 and 5 should cover dialysis modalities and tunneled vascular access options, emphasizing the long-term benefits of AVF [45]. Additionally, equitable clinical decision making and tailored interventions, such as educational programs for both patients and providers, are essential to reinforcing the benefits of AVF across all demographic groups [43]. Future research should explore whether vascular access selection patterns reflect clinical necessity or systemic inequities and further investigate the impact of socioeconomic status and healthcare access on decision making.

Education on the importance of arteriovenous fistula (AVF) inclusion in vascular access decisions is crucial, particularly as patients may not fully understand its long-term benefits. In some cases, the decision to forego AVF is influenced by convenience, lack of adequate information, or misconceptions about the procedure [46,47]. Additionally, family members, who often accompany patients to consultations, play a significant role in the decision-making process. Their perceptions and concerns, whether based on limited knowledge or cultural factors, can impact the patient’s choices regarding vascular access options. Therefore, comprehensive education that includes both patients and their families is essential to ensure that AVF is considered as a primary option for vascular access, empowering patients to make informed decisions that optimize their long-term health outcomes [48].

Interdisciplinary care has emerged as an effective alternative to traditional nephrology care, enhancing patient education, health outcomes, and tunneled access placement. Studies from the United States, Taiwan, and Canada demonstrate that multidisciplinary care improves vascular access outcomes. Patients in interdisciplinary CKD clinics, who were more likely to start dialysis with tunneled vascular access rather than a catheter [49,50], had lower CVC use [51] and experienced fewer urgent dialysis starts [52]. These findings highlight the importance of team-based approaches in optimizing vascular access and patient outcomes. It is important to note that patient preferences and physician guidance play a significant role in the selection of vascular access and can influence survival outcomes [44]. Patients often make decisions based on personal factors, such as previous experiences, perceived quality of life, and the potential risks and benefits of each access type [53]. For example, some patients may prefer a certain type of access due to concerns about comfort, long-term care, or aesthetic considerations [17]. On the other hand, physicians, based on their clinical expertise, guide patients toward the most appropriate vascular access choice considering individual factors such as comorbidities, anatomy, and expected complications [44]. The alignment of patient preferences with physician recommendations in regard to the sociocultural environment of the patient can positively impact adherence and satisfaction, which, in turn, may enhance the success of the chosen vascular access [54]. A more in-depth exploration of these factors could provide valuable insights into how decision-making processes, influenced by both patient preferences and physician guidance, affect long-term outcomes in hemodialysis patients. In addition to this, it is important to take care of the pre-ESRD care as a key factor in vascular access decisions. It is important to note that in Mexico, this service is typically available within hemodialysis units. In our study, all patients, regardless of vascular access type, received the same pre-ESRD planning, health education, and guidance before requiring hemodialysis. As IMSS beneficiaries in Colima, they were treated at the same medical center, minimizing differences in outcomes due to prior education. However, our study did not include specific pre-ESRD care data. While this factor could influence access decisions, the institution lacked a dedicated educational program for kidney disease, representing an area for improvement around the world [55]. Previous reports highlight delays in implementing a national renal health program in Mexico [56]. Our research focused on vascular access outcomes in hemodialysis patients, but future studies should explore the impact of pre-ESRD care on access selection and prognosis.

Our study has several limitations. First, as with all observational studies, patients were not randomly assigned to different vascular access groups, making the study susceptible to selection bias. Patients typically receive vascular access based on clinical decisions, which may be influenced by their individual characteristics, such as comorbidities or disease severity. This can lead to differences in outcomes that are not solely attributable to the type of vascular access but, rather, to the underlying health status of the patients. Despite adjusting for multiple covariates, residual confounding cannot be entirely ruled out. Second, another limitation is the single-center nature of this study, which may restrict the generalizability of our findings to other healthcare settings with different resources, patient demographics, or clinical practices. Future multicenter studies with more diverse populations are needed to validate these results and provide a broader understanding of vascular access selection and outcomes. Third, we lacked formal assessments of patient preferences and clinical staff proficiency, preventing us from exploring their potential influence on vascular access selection and long-term outcomes. Future research incorporating qualitative methodologies could offer valuable insights into how patient perceptions, health literacy, and clinician decision making impact vascular access choices. Finally, we did not evaluate socioeconomic factors and healthcare accessibility, which could play a significant role in vascular access selection and patient outcomes. Future studies should consider these aspects to provide a more comprehensive perspective.

Notwithstanding these limitations, our study has several notable strengths. The ambispective cohort design, large sample size, and long-term follow-up period enhance the study’s robustness and reliability. The use of multivariate Cox regression analysis allowed for rigorous adjustment for potential confounders, strengthening the validity of our findings. Additionally, our study provides valuable insights into vascular access practices among populations living in under-resourced areas, who remain underrepresented in kidney health research despite being disproportionately affected by poor health outcomes. By examining data from a center specializing in hemodialysis (following the ISMMS model of care), this study contributes important evidence that may help inform future strategies to improve vascular access selection and patient outcomes in hemodialysis patients living in under-resourced areas.

## 5. Conclusions

In conclusion, this study underscores vascular access type as a key determinant of long-term survival in hemodialysis patients, with AVFs providing significant survival benefits over both tunneled and non-tunneled catheters. These findings reinforce the need to prioritize AVFs as the preferred vascular access, emphasizing the importance of early nephrology referral, patient education, and structural interventions to ensure timely AVF placement and maturation. This is particularly critical for older adults and diabetic patients, whose survival may heavily depend on having optimal vascular access. Addressing modifiable risk factors, such as diabetes management and infection prevention, is essential to improving vascular access outcomes in this high-risk population. Future research should focus on optimizing vascular access care, identifying barriers to AVF utilization, and developing strategies to enhance equitable access across diverse healthcare settings. Additionally, further studies are needed to evaluate the impact of multidisciplinary care models in improving vascular access planning and reducing disparities in access selection and outcomes.

## Figures and Tables

**Table 1:** Comparison of key clinical characteristics of hemodialysis patients based on vascular access route.

Variable	All Patients 100% (428)	Catheter			*p*-Value *			
		Fistula A-V 27.8% (119)	Tunneled 53.0% (227)	Temporal 19.2% (82)	Inter-Group	F vs. P	F vs. T	P vs. T
>60 years	52.6% (225)	33.6% (40)	59.5% (135)	61.0% (50)	<0.001	<0.001	<0.001	0.896
Female	38.6% (165)	26.1% (31)	42.7% (97)	45.1% (37)	0.003	0.002	0.006	0.795
Diabetes	59.8% (256)	42.9% (51)	63.4% (144)	74.4% (61)	<0.001	<0.001	<0.001	0.078
Hypertension (HTA)	80.4% (344)	84.0% (100)	80.6% (183)	74.4% (61)	0.245	0.467	0.107	0.269
Hospitalizations	23.6% (101)	13.4% (16)	25.6% (58)	32.9% (27)	0.003	0.009	0.001	0.248
Acute Myocardial Infarction (AMI)	7.2% (31)	9.2% (11)	6.2% (14)	7.3% (6)	0.587	0.382	0.798	0.794
Stroke (EVC)	5.60% (24)	3.40% (4)	5.30% (12)	9.80% (8)	0.168	0.592	0.073	0.190
Sepsis	12.1% (52)	6.7% (8)	15.9% (369	9.8% (8)	0.03	0.017	0.441	0.201
Mortality	65.7% (281)	34.5% (41)	73.6% (167)	89.0% (73)	<0.001	<0.001	<0.001	0.003

* To compare three groups (intergroup analysis), or to compare two groups, Fisher’s exact test was used. F = Fistula A-V. P = Tunneled. T = Temporal.

**Table 2:** Overall survival of hemodialysis patients in 84-month follow-up (7 years), according to vascular access route.

	Survival Rate At			Survival Time		
	2 Years	5 Years	7 Years	Months	95% CI	
Global	70.3%	41.6%	34.3%	49.9	47.0	52.8
Fistula A-V	94.1%	74.8%	65.5%	72.4	68.7	76.2
Tunneled	70.0%	35.2%	26.4%	46.8	43.1	50.6
Non-tunneled	36.6%	11.0%	11.0%	25.9	20.4	31.4
*p*-value intergroup	<0.001	<0.001	<0.001	<0.001		
*p*-value (F vs. P)	<0.001	<0.001	<0.001	<0.001		
*p*-value (F vs. T)	<0.001	<0.001	<0.001	<0.001		
*p*-value (P vs. T)	<0.001	<0.001	<0.001	<0.001		

F = Fistula A-V. P = Tunneled. T = Temporal.

**Table 3:** Omnibus tests of model coefficients.

	Overall (Score)			Change from Previous Step			Change from Previous Block		
−2 Loglikelihood	χ^2^	Df	Sig.	χ^2^	df	Sig.	χ^2^	df	Sig.
2469.930	92.166	8	<0.001	90.201	8	<0.001	90.201	8	<0.001

df: Degrees of freedom; Sig.: Statistical significance.

**Table 4:** Univariate and multivariate cox regression model for overall survival in 7-year follow-up.

	Unadjusted	95% CI			Adjusted	95% CI		
Covariate	HR	Lower	Upper	*p*	HR	Lower	Upper	*p*
Age > 60 years	1.881	1.464	2.418	<0.001	1.345	1.032	1.752	0.028
Female	1.130	0.890	1.435	0.317				
Diabetic	2.428	1.845	3.196	<0.001	2.393	1.784	3.210	<0.001
Hypertension (HTA)	0.549	0.418	0.721	<0.001	0.474	0.358	0.627	<0.001
Hospitalization	1.467	1.134	1.899	0.004	1.654	1.274	2.146	<0.001
Sepsis	1.002	0.715	1.404	0.990				
Acute Myocardial Infarction (AMI)	1.022	0.648	1.613	0.956				
Stroke (EVC)	0.923	0.570	1.495	0.744				

The predictors of significant outcomes were determined (univariate model) (unadjusted HR) and subsequently examined in a multivariable model (adjusted HR). Forward stepwise Cox regression was then used to identify the most efficient model. For the stepwise regression, a variable entry threshold of 0.05 and a removal threshold of 0.10 were applied. The vascular access route (AVF, tunneled catheter, or non-tunneled catheter) was incorporated into the model as “strata”.

**Table 5:** Overall survival during a 7-year follow-up according to the presence or absence of risk factors and the type of vascular access.

Variable (Factor)	Patients (*n*)	Factor Presence	Vascular Access Type			
			Global (*n* = 428)	AVF (*n* = 119)	Tunneled (*n* = 227)	Non-Tunneled (*n* = 82)
>60 years old	203	No	51.20%	73.40%	43.50%	18.80%
	225	Yes	19.10%	50.00%	14.80%	6.00%
*p*-value *			<0.001	0.015	<0.001	0.144
Diabetes	172	No	58.10%	83.80%	47.00%	19.00%
	256	Yes	18.40%	41.20%	14.60%	8.20%
*p*-value *			<0.001	<0.001	<0.001	0.224
Hypertension	84	No	16.70%	36.80%	13.60%	4.80%
	344	Yes	38.70%	71.00%	29.50%	13.10%
*p*-value *			<0.001	0.007	0.036	0.435
Hospitalization	327	No	40.4%	70.9%	30.8%	12.7%
	101	Yes	14.90%	31.30%	13.80%	7.40%
*p*-value *			<0.001	0.004	0.015	0.711

* *p*-value: Comparison of survival rates between “Yes” and “No” for each variable in each vascular access stratum.

**Table 6:** Stratum-specific effects on 7-year mortality risk with the use of tunneled and non-tunneled catheters compared to arteriovenous fistulas.

Factor Presence	No				Yes			
	AdHR	Lower	Upper	*p*	AdHR	Lower	Upper	*p*
All								
AVF	1 (Baseline value)							
Tunneled	2.892	2.036	4.108	<0.001				
Non-tunneled	5.047	3.333	7.643	<0.001				
≥60 years old								
AVF	1 (baseline value)				1 (baseline value)			
Tunneled	2.261	1.344	3.806	0.002	3.132	1.939	5.059	<0.001
Non-tunneled	4.570	2.425	8.613	<0.001	6.276	3.473	11.342	<0.001
Diabetes								
AVF	1 (baseline value)				1 (baseline value)			
Tunneled	3.416	1.734	6.729	<0.001	2.603	1.735	3.905	<0.001
Non-tunneled	10.024	4.351	23.098	<0.001	4.418	2.752	7.093	<0.001
Hypertension								
AVF	1 (baseline value)				1 (baseline value)			
Tunneled	2.618	1.346	5.092	<0.001	2.857	1.896	4.306	<0.001
Non-tunneled	4.319	1.981	9.415	<0.001	5.531	3.380	9.051	<0.001
Hospitalization								
AVF	1 (baseline value)				1 (baseline value)			
Tunneled	2.920	1.940	4.397	<0.001	2.449	1.260	4.761	0.008
Non-tunneled	5.223	3.170	8.606	<0.001	7.814	3.063	19.936	<0.001

Multivariate Cox proportional hazards regression analyses were performed. In different strata (Presence of the Factor: No or Yes), the 7-year mortality risk was calculated for the vascular access route (tunneled catheter or non-tunneled catheter), with AVF as the baseline value (HR = 1). The variables of being 60 years or older, diabetes, hypertension, and a history of hospitalization were included as covariates for all analyses.

## Data Availability

The original contributions presented in the study are included in the article; further inquiries can be directed to the corresponding author.
